# Supplementary material for: Optimization of machine tool processing scheduling based on differential evolution algorithm
Source: PLoS One. 2025 Oct 8;20(10):e0333691. doi: 10.1371/journal.pone.0333691 (PMC12507223; doi:10.1371/journal.pone.0333691)
Supplement: S1 Text — (PDF) [file pone.0333691.s001.pdf]

%%%%%%%%%%%%%%%The main procedure of this article is as follows. Please contact the correspondent if it is complete.

```
%% Clear environment variables
warning off                    % Turn off warning messages
close all                      % Close all open figure windows
clear                          % Clear all variables
clc                            % Clear the command window
tic
% restoredefaultpath
%% Import Data
f = xlsread('daoju.xlsx','Sheet1','A1:A200');
[x, y] = data_process(f, 12);     % Step size is 12

n = size(x, 1);
m = round(n * 0.7);            % Use the first 70% for training, and the last 10% for prediction

P_train_pso = x(1:m,:)';
T_train_pso = y(1:m,:)';

P_test_pso = x(m+1:end,:)';
T_test_pso = y(m+1:end,:)';

f_ = size(P_train_pso, 1);                 % Input feature dimension
outdim = 1;                                % Output is the last column

%% Data Normalization
```

```
[p_train_pso, ps_input] = mapminmax(P_train_pso, 0, 1);
p_test_pso = mapminmax('apply', P_test_pso, ps_input);

[t_train_pso, ps_output] = mapminmax(T_train_pso, 0, 1);
t_test_pso = mapminmax('apply', T_test_pso, ps_output);

%% Divide into Training and Testing Sets
M = size(P_train_pso, 2);
N = size(P_test_pso, 2);

%% Optimization Algorithm Parameter Settings
SearchAgents_no = 8;                    % Number of particles
Max_iteration = 15;                     % Maximum number of iterations
dim = 3;                                % Number of optimization parameters
lb = [1e-3, 10, 1e-4];                  % Lower bounds for parameters (learning rate, hidden layer nodes, regularization coefficient)
ub = [1e-2, 30, 1e-1];                  % Upper bounds for parameters (learning rate, hidden layer nodes, regularization coefficient)

fitness = @(x)fical(x,p_train_pso,t_train_pso,f_);

[Best_score, Best_pos, Convergence_curve] = PSO(SearchAgents_no, Max_iteration, lb, ub, dim, fitness);

%% Record Best Parameters
Best_pos(2) = round(Best_pos(2));
best_lr = Best_pos(1, 1);
best_hd = Best_pos(1, 2);
```

```
best_l2 = Best_pos(1, 3);


%% Build Model
% ---------------------- Modify the model structure when adjusting the model structure in fical.m -------------------------
layers = [
    sequenceInputLayer(f_)              % Input layer


    lstmLayer(best_hd)                  % LSTM layer
    reluLayer                           % ReLU activation layer


    fullyConnectedLayer(outdim)         % Output regression layer
    regressionLayer];


%% Parameter Settings
% ---------------------- Modify model parameters when adjusting the model parameters in fical.m -------------------------
options_pso = trainingOptions('adam', ...             % Adam gradient descent algorithm
         'MaxEpochs', 2500, ...                        % Maximum number of epochs 500
         'InitialLearnRate', best_lr, ...          % Initial learning rate best_lr
         'LearnRateSchedule', 'piecewise', ...    % Learning rate decay
         'LearnRateDropFactor', 0.5, ...            % Learning rate decay factor 0.1
         'LearnRateDropPeriod', 400, ...            % Learning rate will be reduced by a factor of 0.5 every 400 epochs
         'Shuffle', 'every-epoch', ...               % Shuffle the dataset every epoch
         'ValidationPatience', Inf, ...             % Disable validation
         'L2Regularization', best_l2, ...           % Regularization parameter
```

```
        'Plots', 'training-progress', ...      % Display progress plots
        'Verbose', false);

%% Train the Model
net_pso = trainNetwork(p_train_pso, t_train_pso, layers, options_pso);

%% Simulation Verification
t_sim1_pso = predict(net_pso, p_train_pso);
t_sim2_pso = predict(net_pso, p_test_pso);

%% Data Reverse Normalization
T_sim1_pso = mapminmax('reverse', t_sim1_pso, ps_output);
T_sim2_pso = mapminmax('reverse', t_sim2_pso, ps_output);
T_sim1_pso = double(T_sim1_pso);
T_sim2_pso = double(T_sim2_pso);

%% Root Mean Square Error
error1_pso = sqrt(sum((T_sim1_pso - T_train_pso).^2) ./ M);
error2_pso = sqrt(sum((T_sim2_pso - T_test_pso ).^2) ./ N);
toc

%% Plot
figure
plot(1 : length(Convergence_curve), Convergence_curve, 'linewidth', 1.5);
title('PSO-LSTM', 'FontSize', 10);
xlabel('Iteration', 'FontSize', 10);
ylabel('Fitness Value (MSE)', 'FontSize', 10);
```

```
grid off

%% Test Set Results
figure;
plotregression(T_test_pso, T_sim2_pso, ['Regression Plot']);
figure;
ploterrhist(T_test_pso - T_sim2_pso, ['Error Histogram']);

%% Root Mean Square Error RMSE
error1_pso = sqrt(sum((T_sim1_pso - T_train_pso).^2)./M);
error2_pso = sqrt(sum((T_test_pso - T_sim2_pso).^2)./N);

%% Coefficient of Determination
R1 = 1 - norm(T_train_pso - T_sim1_pso)^2 / norm(T_train_pso - mean(T_train_pso))^2;
R2 = 1 - norm(T_test_pso - T_sim2_pso)^2 / norm(T_test_pso - mean(T_test_pso))^2;

%% Mean Squared Error MSE
mse1 = sum((T_sim1_pso - T_train_pso).^2)./M;
mse2 = sum((T_sim2_pso - T_test_pso).^2)./N;

%% RPD (Remaining Prediction Residuals)
SE1 = std(T_sim1_pso - T_train_pso);
RPD1 = std(T_train_pso) / SE1;

SE = std(T_sim2_pso - T_test_pso);
RPD2 = std(T_test_pso) / SE;
```

```
%% Mean Absolute Error MAE
MAE1 = mean(abs(T_train_pso - T_sim1_pso));
MAE2 = mean(abs(T_test_pso - T_sim2_pso));

%% Mean Absolute Percentage Error MAPE
MAPE1 = mean(abs((T_train_pso - T_sim1_pso)./T_train_pso));
MAPE2 = mean(abs((T_test_pso - T_sim2_pso)./T_test_pso));

%% Training Set Plot
figure
%plot(1:M,T_train,'r-*',1:M,T_sim1,'b-o','LineWidth',1)
plot(1:M, T_train_pso, 'r-', 1:M, T_sim1_pso, 'b-', 'LineWidth', 1.5)
legend('True Values', 'PSO-LSTM Predicted Values')
xlabel('Prediction Sample')
ylabel('Prediction Result')
string = {'Training Set Prediction Comparison'; ['(R^2 = ' num2str(R1) ' RMSE = '
num2str(error1_pso) ' MSE = ' num2str(mse1) ' RPD = ' num2str(RPD1) ')']};
title(string)

%% Training Set Error Plot
figure
%plot(1:M,T_train-M,T_sim1,'','LineWidth',1)
plot(1:M, T_train_pso - T_sim1_pso, 'k', 'LineWidth', 1.5)
legend('Training Set Error')
xlabel('Prediction Sample')
ylabel('Error')
```

```
%% Prediction Set Plot

figure

plot(1:N, T_test_pso, 'r-', 1:N, T_sim2_pso, 'b-', 'LineWidth', 1.5)

legend('True Values', 'PSO-LSTM Predicted Values')

xlabel('Prediction Sample')

ylabel('Prediction Result')

string = {'Test Set Prediction Comparison'; ['(R^2 = ' num2str(R2) ' RMSE = '
num2str(error2_pso) ' MSE = ' num2str(mse2) ' RPD = ' num2str(RPD2) ')']};

title(string)


%% Test Set Error Plot

figure

ERROR3 = T_test_pso - T_sim2_pso;

plot(T_test_pso - T_sim2_pso, 'b-*', 'LineWidth', 1.5)

xlabel('Test Set Sample Number')

ylabel('Prediction Error')

title('Test Set Prediction Error')

grid on;

legend('PSO-LSTM Prediction Output Error')


%% Linear Fitting Plot

%% Training Set Fitting Plot

figure

plot(T_train_pso, T_sim1_pso, '*r');

xlabel('True Values')

ylabel('Predicted Values')

string = {'Training Set Fitting'; ['R^2_c = ' num2str(R1) ' RMSEC = '
```

```
num2str(error1_pso)]};
title(string)
hold on;
h = lsline;
set(h, 'LineWidth', 1, 'LineStyle', '-', 'Color', [1 0 1])

%% Prediction Set Fitting Plot
figure
plot(T_test_pso, T_sim2_pso, 'ob');
xlabel('True Values')
ylabel('Predicted Values')
string1 = {'Test Set Fitting'; ['R^2_p = ' num2str(R2) ' RMSEP = ' num2str(error2_pso)]};
title(string1)
hold on;
h = lsline();
set(h, 'LineWidth', 1, 'LineStyle', '-', 'Color', [1 0 1])

%% Calculate Average
R3 = (R1 + R2) / 2;
error3 = (error1_pso + error2_pso) / 2;

%% Overall Data Linear Prediction Fitting Plot
tsim = [T_sim1_pso, T_sim2_pso]';
S = [T_train_pso, T_test_pso]';
figure
plot(S, tsim, 'ob');
xlabel('True Values')
```

```
ylabel('Predicted Values')
string1 = {'All Sample Fitting Prediction'; ['R^2_p = ' num2str(R3) ' RMSEP = '
num2str(error3)]};
title(string1)
hold on;
h = lsline();
set(h, 'LineWidth', 1, 'LineStyle', '-', 'Color', [1 0 1])

%% Display Evaluation Metrics
disp('----------------------- Error Calculation -------------------------')
disp('The evaluation results are as follows:')
disp(['Mean Absolute Error (MAE) = ', num2str(MAE2)])
disp(['Mean Squared Error (MSE) = ', num2str(mse2)])
disp(['Root Mean Square Error (RMSEP) = ', num2str(error2_pso)])
disp(['Coefficient of Determination (R^2) = ', num2str(R2)])
disp(['Remaining Prediction Residual (RPD) = ', num2str(RPD2)])
disp(['Mean Absolute Percentage Error (MAPE) = ', num2str(MAPE2)])
grid

%%%%%%%%%%%%%PSO
function [gbest, g, Convergence_curve] = PSO(N, T, lb, ub, dim, fobj)
%% Define Particle Swarm Optimization parameters
% N - population size, T - number of iterations
%% Randomly initialize the population
D = dim;                        % Particle dimension
c1 = 1.5;                     % Learning factor 1
c2 = 1.5;                     % Learning factor 2
```

```
w = 0.8;                    % Inertia weight

Xmax = ub;                    % Maximum position value
Xmin = lb;                   % Minimum position value
Vmax = ub;                    % Maximum velocity value
Vmin = lb;                   % Minimum velocity value
%%
%%%%%%%%%%%%%%% Initialize population individuals (limit position and
velocity) %%%%%%%%%%%%%%%

x = rand(N, D).*(Xmax - Xmin) + Xmin;
v = rand(N, D).*(Vmax - Vmin) + Vmin;
%%%%%%%%%%%%%%%%%%% Initialize personal best position and value %%%%%%%%%%%%%%%%%%%
p = x;
pbest = ones(N, 1);
for i = 1:N
    pbest(i) = fobj(x(i, :));
end
%%%%%%%%%%%%%%%%%%%% Initialize global best position and value %%%%%%%%%%%%%%%%%%%%
g = ones(1, D);
gbest = inf;
for i = 1:N
    if (pbest(i) < gbest)
        g = p(i, :);
        gbest = pbest(i);
    end
end
```

```
%%%%%%%%%%% Iterate according to the formula until precision is met or the number of
iterations is reached %%%%%%%%%%%%%

for i = 1:T

    i

    for j = 1:N

        %%%%%%%%%%%%%% Update individual best position and
value %%%%%%%%%%%%%%%%%%

        if (fobj(x(j, :))) < pbest(j)

            p(j, :) = x(j, :);

            pbest(j) = fobj(x(j, :));

        end

        %%%%%%%%%%%%%%%% Update global best position and
value %%%%%%%%%%%%%%%%%%%

        if pbest(j) < gbest

            g = p(j, :);

            gbest = pbest(j);

        end

        %%%%%%%%%%%%%%%%% Update position and velocity
values %%%%%%%%%%%%%%%%%%%

        v(j, :) = w * v(j, :) + c1 * rand * (p(j, :) - x(j, :)) + c2 * rand * (g - x(j, :));

        x(j, :) = x(j, :) + v(j, :);

        %%%%%%%%%%%%%%%%%%%% Boundary condition
handling %%%%%%%%%%%%%%%%%%%%%%%

        if length(Vmax) == 1

            for ii = 1:D

                if (v(j, ii) > Vmax) | (v(j, ii) < Vmin)

                    v(j, ii) = rand * (Vmax - Vmin) + Vmin;

                end
```

```
                if (x(j, ii) > Xmax) | (x(j, ii) < Xmin)
                    x(j, ii) = rand * (Xmax - Xmin) + Xmin;
                end
            end
        else
            for ii = 1:D
                if (v(j, ii) > Vmax(ii)) | (v(j, ii) < Vmin(ii))
                    v(j, ii) = rand * (Vmax(ii) - Vmin(ii)) + Vmin(ii);
                end
                if (x(j, ii) > Xmax(ii)) | (x(j, ii) < Xmin(ii))
                    x(j, ii) = rand * (Xmax(ii) - Xmin(ii)) + Xmin(ii);
                end
            end
        end

    end
    %%%%%%%%%%%%%%%%%%%% Record the global best value at each
iteration %%%%%%%%%%%%%%%%%%%%
   Convergence_curve(i) = gbest; % Record the fitness value of the training set
   disp(['current iteration is: ', num2str(i), ', best fitness is: ', num2str(gbest)]);

end
```
