# Supplementary material for: Optimization of machine tool processing scheduling based on differential evolution algorithm
Source: PLoS One. 2025 Oct 8;20(10):e0333691. doi: 10.1371/journal.pone.0333691 (PMC12507223; doi:10.1371/journal.pone.0333691)
Supplement: Attachment — Submitted filename: Reviewers Comments PONE-D-25-27253.pdf [file pone.0333691.s002.pdf]

Title: Optimization of machine tool processing scheduling based on differential evolution Algorithm.

Manuscript ID: PONE-D-25-27253

Article type: Research article

PLOS ONE

# Reviewer's Comments:

1) It would be beneficial to separate the introduction section from the related work. Additionally, critically analyze individual related papers that are closely aligned with your proposed method. Highlight their strengths and weaknesses, particularly in addressing the specific problem, to clearly demonstrate how the research gap was identified.
2) Revise the methodology section by including a block diagram or flowchart that clearly illustrates the DE algorithm and highlights the hybrid components constituting your main contribution. Keep the description of the standard DE algorithm concise, focusing more on your proposed modifications.
3) Subsection 3.4, which discusses the parameter settings of the DE algorithm, should be moved under the "Experiments and Results" section. Clearly describe the experimental platform and the corresponding parameter configurations.
4) Present the experimental results in tabular form, including the evaluation metrics used. Supplement the tables with appropriate statistical visualizations and provide a thorough discussion of the results.
5) Clearly elaborate on the adaptation of the DE algorithm to discrete scheduling tasks through custom encoding and decoding schemes, emphasizing this as a key contribution of your work.
6) It would strengthen the practical relevance of your proposed method if it were validated using a real-world industrial dataset. Additionally, focus on conducting a deeper analysis of the experimental results.
7) The multiple objectives mentioned in the manuscript appear to be unnecessary, as the main focus of this work is on profit maximization as a single objective.
8) Simplify the assumptions made in relation to the main objective. The model currently depends on simplifications such as fixed switching times, idealized precedence constraints, and homogeneous job sizes, which may limit its applicability in complex manufacturing environments.
9) To better demonstrate the superiority of your proposed method, consider benchmarking your DE-based approach against modern metaheuristics such as NSGA-II, MOEA/D, and ACD.
10) Further discussion on the empirical performance of the proposed method in handling complex scheduling instances; particularly in the context of manufacturing would significantly strengthen your paper.
