# Supplementary material for: Optimization of machine tool processing scheduling based on differential evolution algorithm
Source: PLoS One. 2025 Oct 8;20(10):e0333691. doi: 10.1371/journal.pone.0333691 (PMC12507223; doi:10.1371/journal.pone.0333691)
Supplement: Attachment — Submitted filename: Reviewers Comments PONE-D-25-27253-R1.pdf [file pone.0333691.s005.pdf]

Title: Optimization of machine tool processing scheduling based on differential evolution Algorithm.

Manuscript ID: PONE-D-25-27253_R1

Article type: Research article

PLOS ONE

Reviewer's Comments:

1) It is quite impressive that the authors have substantially addressed the issues raised in the reviewers' comments on the original manuscript. Their revisions have significantly improved the quality of the manuscript, bringing it up to the standard required by this reputable journal. I therefore confidently recommend that the manuscript be accepted for publication.
2) You might find these papers useful, and citing them could strengthen your manuscript:
   http://doi.org/10.1049/wss2.12100
   https://doi.org/10.1016/j.iswa.2024.200381
   https://doi.org/10.1016/j.iswa.2024.200442
