# Supplementary material for: Optimization of machine tool processing scheduling based on differential evolution algorithm
Source: PLoS One. 2025 Oct 8;20(10):e0333691. doi: 10.1371/journal.pone.0333691 (PMC12507223; doi:10.1371/journal.pone.0333691)
Supplement: Attachment — Submitted filename: REVIEW.docx [file pone.0333691.s003.docx]

1. HEADING 2 have no content, need to add at least a paragraph under this heading
2. Heading 2.2 , the adjustment of variables’ was done on what basis, either add explanation or include citation
3. Heading 3 also have no content, just like heading 2
4. In figure 2 the “feedback loop” must come after the last block “G=G+1
5. FOR THE HEADING “3.4 Parameters setting of DE algorithm” reference/citation is missing for their value selection
6. Figure 3 must be more explanatory, should show the local maxima and minima in the fig
7. Lines 395 and 398 , what examples? Are they examples or your contribution? If it’s your then rephrase the sentence.
8. Again at line 404 and next the values of parameters have been selected on what criteria? Kindly specify
9. Figure 4 must be cited in the text and add some explanations about it.
10. Table 1 better to add some discussion on table findings
11. Heading 4.1 again the reference or explanation is required to added for parameters setting
12. Better to add those points, discuss about the achieved tasks, mentioned in gaps section
13. Must add the recent references, the latest reference, added is from year 2021
